# Validation and psychometric evaluation of the French version of the recovery experience questionnaire: internal consistency and validity assessment

**DOI:** 10.3389/fpsyg.2024.1466905

**Published:** 2024-10-02

**Authors:** Mathieu Le Moal, Roy Thurik, Olivier Torrès

**Affiliations:** ^1^LabEx Entreprendre, MRM, Université de Montpellier - MOMA, Montpellier, France; ^2^Laboratoire EPSYLON, Université Paul-Valery, Montpellier, France; ^3^LabEx Entreprendre, MBS School of Business, Montpellier, France; ^4^Erasmus School of Economics, Rotterdam, Netherlands

**Keywords:** recovery experience, France, health, stress, factor analysis, scale validation, entrepreneur, psychometrics

## Abstract

**Background:**

Entrepreneurs often experience high levels of stress, anxiety, and burnout due to the demanding nature of their professional activities. Therefore, recovery from work-related stress is a relevant activity for entrepreneurs. The Recovery Experience Questionnaire (REQ) is a widely used 16-item self-reported measure covering four recovery factors: psychological detachment from work, relaxation, mastery, and control. The present study addresses the validation of a French version of the REQ.

**Methods:**

A total of 1,043 French entrepreneurs from various sectors participated in this study. Internal consistency and correlations were examined to assess the psychometric properties of the French version of the REQ. Confirmatory factor analysis (CFA) was used to validate the four-factor structure of the REQ, with seven error covariances added to improve model fit.

**Results:**

The French version of the REQ demonstrated good internal consistency (psychological detachment: *α* = 0.88, relaxation: α = 0.91, mastery: α = 0.90, control: α = 0.91). CFA supported that the four-factor structure was confirmed based on the following data: RMSEA = 0.071 (95% CI [0.066, 0.077]), CFI/TLI = 0.955/0.950, SRMR = 0.050, and χ^2^ (108) = 593.861, *p* < 0.001. Significant correlations were found between REQ scores and health indicators such as stress, loneliness, physical health, mental health, and sleep quality. The results confirm that the REQ is a valid and reliable measure for assessing recovery experiences among French entrepreneurs.

**Conclusion:**

We conclude that the REQ is a valid measure and a useful tool for research on entrepreneurs’ general health. Additionally, the validated French version of the REQ can be applied to other working populations, making it a versatile instrument for evaluating health and recovery in diverse occupational settings. To support this claim, we conducted the same validation analysis on a sample of 1,231 French agricultural employees, again showing that REQ is a valid and reliable measure for assessing recovery experiences.

## 1 Introduction

Phrases such as “I do not have time to be sick” or “I only get sick when I am on vacation” are common among entrepreneurs^1^. Nevertheless, over 45% of entrepreneurs report that their workdays are stressful (Torrès, 2017). According to Cardon et al. (2009), entrepreneurs often neglect their own needs, prioritizing business development at the expense of their health. The health of entrepreneurs in the workplace is a subject that has been underresearched (Stephan, 2018; Vinberg et al., 2012). While entrepreneurship can be beneficial for personal and professional growth (Williamson et al., 2021) and can have beneficial effects (Torrès and Thurik, 2019), many entrepreneurs regularly face excessive workloads, pressure (Lechat and Torrès, 2017), and high levels of stress (Wach et al., 2021; Williamson et al., 2021), which can lead to burnout (Torrès and Kinowski-Moysan, 2019). When an entrepreneur can no longer perform his or her duties, the entire business is at risk (Torrès and Thurik, 2019). These findings highlight the importance and necessity of addressing entrepreneurs’ health. Positive psychological constructs play a crucial role in promoting mental well-being (Vancappel et al., 2022). In other words, the stress factors associated with entrepreneurship do not necessarily have to dominate, as the physiological and psychological resources spent in entrepreneurship can be partially restored through recovery (Karabinski et al., 2021; Sonnentag, 2018b). Recovery reduces the harmful impact of stress on the body and mind, leading to increased health and productivity (Williamson et al., 2019). However, mentally detaching from stressful work and finding time for recovery activities is particularly challenging for entrepreneurs (Williamson et al., 2021) who typically work very long hours, often including evenings and weekends (Eurostat, 2021).

Given the significant stress burden on entrepreneurs, the need for validated tools to assess recovery in this population is crucial. The Recovery Experience Questionnaire (REQ), which has been validated in multiple languages and cultural contexts, is an ideal instrument for this purpose. Validating the Recovery Experience Questionnaire (REQ) in French ensures its applicability in a significant linguistic context and enhances research and practical interventions in occupational health. French is spoken by approximately 320 million people globally and is an official language in 29 countries, making it an important language both in terms of number of speakers and the geographical diversity of its use (Eberhard et al., 2022; World Population Review, 2024). Previous validations of the REQ in various languages and contexts, including Spain (Sanz-Vergel et al., 2010), South Korea (Park et al., 2011), Finland (Kinnunen et al., 2011), Japan (Shimazu et al., 2012), South Africa (Mostert and Els, 2015), the Netherlands (Bakker et al., 2015), Sweden (Almén et al., 2018), Peru (Carranza Esteban et al., 2022), Lithuania (Kazlauskas et al., 2023), and Brazil (Pérez-Nebra et al., 2023), highlight the universal relevance and utility of the REQ. However, none of these studies focus on entrepreneurs. Our study is the first to focus on entrepreneurs, who represent an important part of the economy. For instance, there were 4.5 million entrepreneurs in the nonagricultural sector in 2021 (INSEE, 2023), which amounts to 14.95% of the French working population. The objective of the present study is to analyze the psychometric properties of the Recovery Experience Questionnaire (REQ) for French entrepreneurs. And then to do the same for French agricultural employees showing the usefulness of the French version of REQ beyond the area of entrepreneurs. See Table 1 for our new French version and the original English versions of Sonnentag and Fritz (2007). First, we conduct an exploratory factor analysis (EFA) to identify the underlying factor structure of the REQ and to ensure that the items load appropriately on the expected dimensions. Next, we test the structural validity of the four-factor structure of the REQ with the four correlated latent factors of psychological detachment, relaxation, mastery, and control via confirmatory factor analysis (CFA). We also assess the internal consistency of the REQ using McDonald’s Omega and Cronbach’s alpha to evaluate its reliability, ensuring that the items within each factor consistently measure the same underlying construct. Additionally, we explore the associations between the REQ score and perceived stress, perceived loneliness, physical and mental health, and sleep quality in our sample, thus evaluating the concurrent validity of the REQ in relation to these health indicators. We also provide sociodemographic data of our sample for comparison with future questionnaires.

**Table 1:** French version of the recovery experience questionnaire (REQ-F).

Dimensions		French items	English items^a^
Psychological detachment	1	J’oublie le travail	I forget about work
	2	Je ne pense pas du tout au travail	I do not think about work at all
	3	Je me détache de mon travail	I distance myself from my work
	4	Je prends une pause par rapport aux demandes au travail	I get a break from the demands of work
Relaxation	5	Je décompresse et me détends	I kick back and relax
	6	Je fais des choses relaxantes	I do relaxing things
	7	Je prends du temps pour me relaxer	I use the time to relax
	8	Je consacre du temps à mes loisirs	I take time for leisure
Mastery	9	J’apprends de nouvelles choses	I learn new things
	10	Je recherche des défis intellectuels à relever	I seek out intellectual challenges
	11	Je fais des choses qui me challengent	I do things that challenge me
	12	Je fais quelque chose pour élargir mon horizon	I do something to broaden my horizons
Control	13	J’ai l’impression de pouvoir décider quoi faire par moi-même	I feel like I can decide for myself what to do
	14	Je décide de mon emploi du temps	I decide my own schedule
	15	Je choisis moi-même comment je vais passer mon temps	I determine for myself how I will spend my time
	16	Je fais les choses comme je le souhaite	I take care of things the way that I want them done

The items are prefaced with: “Please indicate the extent to which you agree or disagree with the following statements about your activities after your workday.” Responses are scored as follows: 1 = Pas du tout d’accord, 2 = Plutôt pas d’accord, 3 = Ni d’accord ni pas d’accord, 4 = Plutôt d’accord, 5 = Tout à fait d’accord.

a From Sonnentag and Fritz (2007).

## 2 Recovery

Recovery is a term that emerged in the late 20th century and refers to the process through which individuals restore their depleted physical and psychological resources after work (Sonnentag and Fritz, 2007). Work-related recovery, specifically, is the process by which individuals recover from the demands of work to regain their mental and physical health (Sonnentag, 2018b). Research conducted over the past few decades has demonstrated that it is essential to pay attention to recovery processes to understand how general good health can develop, and as well, how work-related health problems can develop (Sonnentag, 2018a). In the current literature, there are two approaches for studying the recovery process (Sonnentag et al., 2022).

The first approach refers to the notion of activity, which initiates the recovery process. However, different types of activities exist, and not all of them allow for recovery. Activities with low-level daily obligations (such as engaging in physical exercise, watching TV and socializing with friends) are sources of well-being and facilitate recovery (Sonnentag et al., 2022), whereas activities with high-level daily obligations (such as house cleaning and childcare) do not facilitate recovery (Steed et al., 2021). This approach helps guide individuals toward various activities for recovery.

The second approach refers to the psychological experience resulting from recovery activities after working hours or during work breaks (Sonnentag et al., 2022). According to Sonnentag and Fritz (2007), four recovery experiences (i.e., psychological detachment, relaxation, mastery, and control) can be distinguished. *First*, psychological detachment from work refers to the experience of mentally distancing oneself from work-related activities. This process involves a disconnection, where the individual avoids thinking about work-related issues or problems. By engaging in such detachment, the demands placed on the individual’s functional systems during working hours are alleviated, allowing for a reduction in affective and self-regulatory resource utilization. *Second*, relaxation corresponds to a reduction in physiological activity through the restoration of the original physiological state and cognitive reevaluation. This allows the mind to refocus on positive affects and reduces the negative affects related to work stress. It occurs during activities intentionally chosen to relax the body and mind, such as meditation, mindfulness, heart coherence exercises, or simply walking in nature while observing beautiful landscapes. *Third*, mastery experiences involve expanding one’s skills outside of professional activities, developing new resources (e.g., a variety of knowledge, better self-understanding), fostering innovation, and encouraging a broader perspective on professional situations. This can be experienced while engaging in stimulating leisure activities that allow learning in areas other than work (e.g., learning a new language, improving in a sport). *Finally*, control during leisure activities constitutes a resource that enables individuals to increase their sense of self-efficacy and competence and to regulate their actions more effectively. This experience occurs when an individual chooses the activity they want to engage in and the timing of the activity.

Thus, without adequate recovery experiences, individuals may experience prolonged stress, which can lead to burnout and negatively affect their overall health (Poulsen et al., 2015). Recovery enables individuals to replenish the mental and physical resources necessary for sustained health, particularly for those in high-demand roles like entrepreneurship.

## 3 Health indicators related to recovery experiences

In the present study, we address stress, loneliness, physical health, mental health, and sleep quality as health indicators. Stress is defined as a state of mental tension or worry resulting from difficult life conditions, where external demands exceed an individual’s resources (Lazarus and Folkman, 1984). Stress is widely recognized as a key factor in the recovery process. Studies have shown that recovery periods can significantly mitigate the negative effects of stress on psychological health (Sonnentag and Fritz, 2007). Specifically, effective recovery reduces cumulative fatigue and stress, thereby promoting better mental balance (Sonnentag and Fritz, 2015). Loneliness is understood as the subjective feeling of social disconnection, occurring when there is a perceived gap between desired and actual social relationships (Hawkley and Cacioppo, 2010). Loneliness at work can significantly impact emotional health, prompting individuals to adopt recovery practices such as psychological detachment to combat emotional exhaustion (Jung et al., 2022). Additionally, practicing mindfulness has been found to reduce loneliness, offering an active recovery method to restore psychological health (Margalit, 2018). Physical health refers to an individual’s state of physical well-being, including the absence of disease or abnormal conditions and the ability to perform daily tasks without undue fatigue or physical impairment (World Health Organization, 1948). Physical health is an essential factor influenced by recovery processes, as stress and burnout can directly affect the body. Mental health is defined as a state of well-being where an individual can realize their abilities, cope with normal life stresses, work productively, and contribute to their community (World Health Organization, 2001). Good mental health is a key outcome of effective recovery, as it helps mitigate the negative impacts of chronic stress and enhances emotional resilience. Sleep quality encompasses the effectiveness of sleep in restoring both physical and mental resources. It includes factors such as sleep duration, the time taken to fall asleep, sleep interruptions, and overall satisfaction with sleep (Buysse et al., 1989). Adequate sleep is crucial for proper recovery, as poor sleep can exacerbate the effects of stress and diminish overall well-being. These aspects are essential for evaluating the general health of small business managers (Guiliani and Torrès, 2018; Wach et al., 2021). We assume a four-factor structure of the REQ scale, as proposed by previous research. Specifically, we hypothesize that the four dimensions of the REQ (psychological detachment, relaxation, mastery, and control) are positively related to physical health, mental health, and sleep quality, and negatively related to stress and loneliness.

Using CFA, Sonnentag and Fritz (2007) compared the proposed four-factor structure to other models, namely, a one-factor model, best-fitting two-factor models, and best-fitting three-factor models. The results showed that the scale items were better represented by four factors than by a single common factor, a two-factor structure, or a three-factor structure. Interestingly, Ginoux et al. (2021) attempted to validate Sonnentag and Fritz's (2007) REQ with a French population and obtained five factors in their CFA and used a 7-point Likert scale instead of the original 5-point scale. Furthermore, other researchers have sought to validate it in French and have reduced the scale from the original 16 items to 12 items (Queiroga et al., 2024). In addition to the study conducted by Sonnentag and Fritz (2007), the REQ has been translated into several languages and validated in different countries. This multitude of validations demonstrates the strong interest and utility of this scale worldwide, thus highlighting the importance of validating it in France. Considering the variety of contrasting models, all the above studies support the four-factor structure proposed by Sonnentag and Fritz (2007). We acknowledge the work of Ginoux et al. (2021) who attempted to validate the REQ using a French survey and found a five-factor structure using a 7-point Likert scale instead of the original 5-point scale. Additionally, Queiroga et al. (2024) sought to validate the REQ in French by reducing the scale from the original 16 items to 12 items. However, we chose to adhere to the original 16-item, 4-factor structure with a 5-point Likert scale as proposed by Sonnentag and Fritz (2007). This decision was driven by the wish to maintain the comparability of findings across international studies and to preserve the integrity of the original model. By retaining the original structure, we aimed to contribute to the REQ’s applicability across cultural and professional contexts, without introducing modifications that could alter the interpretation of the scale. Furthermore, our study specifically adds to the literature by validating the REQ within a population of French entrepreneurs, and also of French agricultural employees.

## 4 Methods

### 4.1 Translation

First, the English version of the REQ was translated into French by a native French-speaking researcher who is fluent in English. Next, a back-translation into English was performed by an English specialist who had not seen the original items. We compared the English and back-translated versions and created a preliminary French version after making a few corrections to the wording, meaning, and content of each item.

### 4.2 Participants

Our sample consists of 1,043 respondents from four surveys: affiliates of the Chamber of Trades and Crafts (CMA30), affiliates of the Amarok network, clients of the insurance company AG2R La Mondiale, and affiliates of the occupational health service AIPALS. The Chambre de Métiers et de l’Artisanat du Gard (CMA30) was created to help craftsmen in their business management and supports them through the social security system for the self-employed. In 2021, CMA30 had 26,837 active craft businesses. Participants from CMA30 were surveyed through an online questionnaire sent in April 2021. The Amarok Observatory is an independent association involved in the study of the physical and mental health of nonsalaried workers, including owners/managers of small and medium-sized businesses, independent traders, liberal professions, and craftsmen. Participants from Amarok were surveyed in November 2021. AG2R La Mondiale is a French not-for-profit social protection and asset management organization, whose governance is based on parity and mutualism. It provides insurance for 15 million individual clients and 500,000 businesses. Participants from AG2R La Mondiale were surveyed in January 2022. Finally, AIPALS is an occupational health service that advises and supports company managers and employees in Montpellier, France, to improve their working conditions and preserve their health throughout their working life. Participants from AIPALS were surveyed in January 2022. Only responses from completed questionnaires were retained, and responses from individuals who (almost) systematically responded with the same value to a large number of questions were eliminated. In total, we retained 360 responses from the CMA30 sample, 345 responses from the AG2R La Mondiale sample, 251 responses from the Amarok sample, and 87 responses from the AIPALS sample. The sociodemographic characteristics of the sample are presented in Table 2. The average age of the sample is 50 years (SD = 9.7), and the majority are men (51.2%). A majority of the respondents (61.8%) were in a relationship. The average experience in the sample was 13.7 years as an entrepreneur, and approximately half of the sample (55%) had a workload of over 50 h per week. The percentage of businesses in manufacturing, including food processing, construction, industry and agriculture, was 17.1%, whereas that in services, including transport, business services, commerce, hospitality, personal services and remaining businesses, was 82.9%. The majority of businesses (81.9%) were small, with fewer than 10 employees, while 18.1% of the businesses had more than 10 employees.

**Table 2:** Descriptive statistics of sociodemographic variables (means, frequencies, standard deviations, percentages).

	*N*	(%)	Mean	(SD)
Age (years)	1,036		50.0	(9.7)
**Gender**				
0 - Male	534	(51.2)		
1 - Female	509	(48.8)		
**Life partner**				
0 - Yes	639	(61.7)		
1 - No	396	(38.3)		
**Education level**				
1 - Self-taught	32	(3.1)		
2 - Vocational training certificate	220	(21.1)		
3 - High school diploma	207	(19.8)		
4 - Associate/Bachelor’s degree	262	(25.1)		
5 - Master’s degree	217	(20.8)		
6 - Doctorate or higher	105	(10.1)		
**Sector of activity**				
0 - Manufacturing	178	(17.1)		
1 - Services	865	(82.9)		
Experience (years)	1,036		13.7	(9.2)
**Weekly workload**				
1–40 h or less	100	(14.6)		
2 - Between 40 and 50 h	207	(30.3)		
3 - Between 50 and 60 h	186	(27.2)		
4 - Between 60 and 70 h	118	(17.3)		
5 - More than 70 h	72	(10.5)		
**Business size**				
0 - Less than 10 employees	854	(81.9)		
1 - More than 10 employees	189	(18.1)		

### 4.3 Measures

#### 4.3.1 Recovery experiences

The REQ (Sonnentag and Fritz, 2007) distinguishes four dimensions (psychological detachment, relaxation, mastery, and control), each measured with four items (questions). The items are prefaced with “Please indicate the extent to which you agree or disagree with the following statements about your activities after your workday. An example item for psychological detachment is “I forget about work.” An example item for relaxation is “I unwind and relax.” An example item for mastery is “I learn new things.” An example item for control is “I decide my own schedule.” Responses are scored as follows: 1 = *Strongly disagree*; 2 = *Somewhat disagree*; 3 = *Neither agree nor disagree*; 4 = *Somewhat agree*; 5 = *Strongly agree*. Scores for each dimension are obtained by averaging the responses to the four questions.

#### 4.3.2 Potential indicators of recovery experiences

##### 4.3.2.1 Perceived stress

Inspired by the single item validated by Houdmont et al. (2019), the stress experienced by the surveyed entrepreneurs was assessed using the following question: “In the past month, would you say that most of your days were 1 = *Not at all stressful*; 2 = *Not very stressful*; 3 = *A little stressful*; 4 = *Quite stressful*; 5 *Extremely stressful*.”

##### 4.3.2.2 Loneliness at work

The loneliness experienced in the workplace was assessed via the following question: “In the past month, in your role as an entrepreneur, did you feel 1 = *Very surrounded;* 2 = *Somewhat surrounded;* 3 = *Neither surrounded nor lonely;* 4 = *Somewhat lonely;* 5 = *Very lonely*.” See Fernet et al. (2016), which was inspired by Stephens et al. (2011) and Stickley et al. (2013).

##### 4.3.2.3 Perceived health

Perceived health was assessed via three 1 item—dimensions, i.e., mental health, physical health, and sleep quality—over the past month, all of which were measured on a 5-point scale ranging from 1 = *Poor*; 2 = *Fair*; 3 = *Good*; 4 = *Very good*; 5 = *Excellent*. These self-assessment dimensions are similar to those collected in large national and international surveys, such as the World Value Survey, the European Value Survey (Mansyur et al., 2008), the National Health and Nutrition Examination Survey in the United States, and the SHARE in Europe. These surveys include the SF-36 survey instrument (Ware and Gandek, 1998) and are recommended for health surveys (Robine et al., 2008). Physical health was measured with one item adapted from Heyman and Jeffers (1963): “During the last month, would you say that your physical health was.” Mental health, adapted from Friedsam and Martin (1963), was assessed with the following item: “During the last month, would you say that your mental health was.” Sleep quality was measured via an item based on the Pittsburgh Sleep Quality Index (PSQI) from Buysse et al. (1989): “During the last month, would you say that your sleep quality was.”

### 4.4 Analyses

We performed a variety of analyses via RStudio (version 2024.04.2 + 764). Initially, exploratory factor analyses were conducted with the 16 items via the unweighted least squares method. Factors were extracted on the basis of eigenvalues greater than one, following the Kaiser rule (Braeken and Van Assen, 2017). Factor loadings were considered acceptable if they exceeded 0.30 (Costello and Osborne, 2005), and communalities were deemed satisfactory if they were above 0.40 (Hair et al., 2010; Tabachnick and Fidell, 2019). For the confirmatory factor analysis (CFA), we adhered to Tanaka’s (1987) suggestion that a significant χ2 should not automatically result in model rejection. Instead, model fit was evaluated via the comparative fit index (CFI) and the Tucker–Lewis index (TLI), with values above 0.90 indicating an acceptable fit and values above 0.95 indicating an excellent fit, and the root mean square error of approximation (RMSEA), with values below 0.06 signifying a good model fit (Browne and Cudeck, 1992; Gana and Broc, 2018). We also considered the χ^2^/df ratio, with values above two indicating a good fit (Byrne, 2016). Promax rotation was then used to obtain factor structures. A CFA was subsequently performed to compare the fit of a one-factor model where all the items measure a general factor of recovery experiences, against a four-factor model, where each item loads onto a specific hypothesized factor (Hurley et al., 1997). Construct validity was evaluated by examining the relationships between recovery experiences and health indicators. Internal consistency was assessed via Cronbach’s alpha, with values above 0.80 indicating good reliability (Cronbach, 1965). Following recent guidelines, McDonald’s Omega was also used to complement this evaluation (Hayes and Coutts, 2020), as it accounts for the unequal sensitivity across items that can affect Cronbach’s alpha. We further examined interitem correlations and conducted correlational analyses to assess the concurrent validity of the REQ scale by exploring its relationship with other constructs.

## 5 Results

### 5.1 Factor structure of the REQ

The means, standard deviations, Cronbach’s alphas, and correlations of the variables used in the study are presented in Supplementary material S1. We assessed interitem correlations and found that all items were significantly correlated with each other, with *p* values less than 0.001. Additionally, items within each dimension showed stronger correlations among themselves than with items from other dimensions (see Table 3). The exploratory factor analysis (EFA) supported a four-factor structure, with eigenvalues greater than one for each factor: relaxation, control, mastery, and psychological detachment (Table 4). Interfactor correlations ranged from 0.27 to 0.61.

**Table 3:** Interitem correlations of the recovery experience questionnaire, French version (REQ-F).

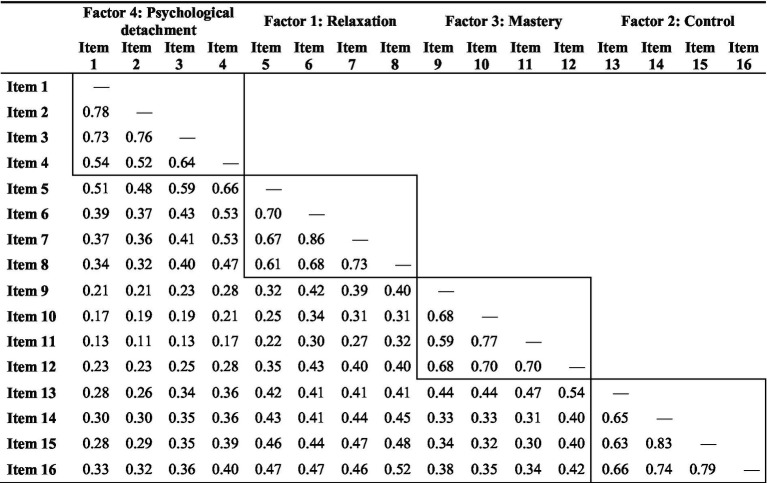

All correlations are significant at *p*-value < 0.001.

**Table 4:** Results of the exploratory factor analysis with unweighted least squares method and promax rotation (*N* = 1,043).

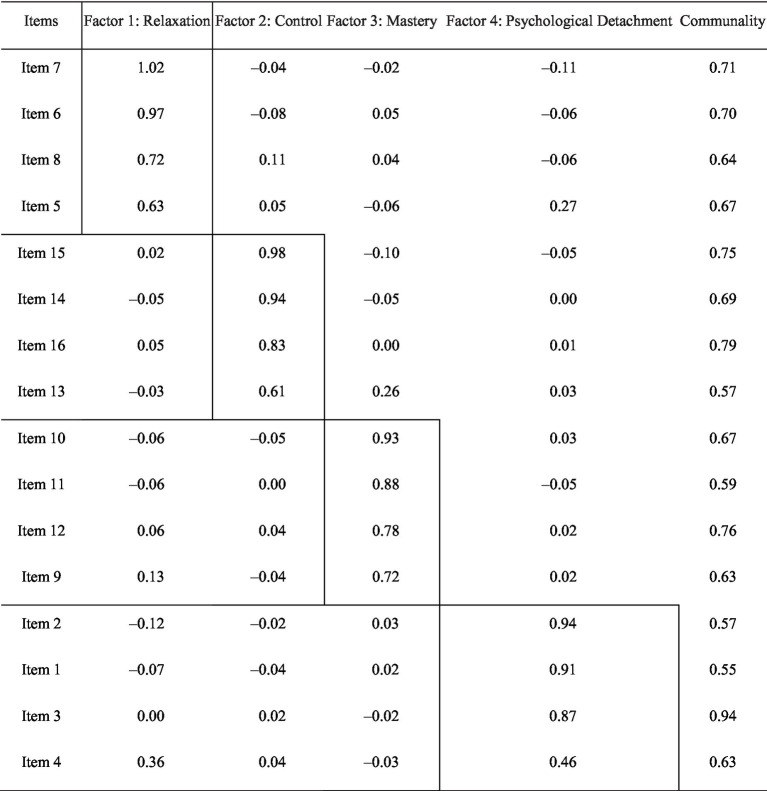

The communality of an item is the proportion of the item’s variance that is explained by the common factors extracted in the model. It is calculated as the sum of the squared factor loadings for a given item across all extracted factors. See Tables 1, 3 for the content and the dimensions of the items.

Initially, the CFA revealed that the correlated four-factor model did not fit the data well, with an RMSEA of 0.088 (95% CI [0.082, 0.093]), CFI/TLI of 0.929/0.925, SRMR of 0.073, χ^2^ (114) = 876.909, *p* < 0.001. Based on high modification indices, seven residual error covariances were added: Detach1 ~ ~ Detach2, Detach3 ~ ~ Detach4, Detach1 ~ ~ Relax2, Relax2 ~ ~ Relax3, Mast2 ~ ~ Mast3, and Contr2 ~ ~ Contr3 (see Supplementary material S2). These modifications address shared variance between certain items, improving model fit without altering the theoretical structure. After these changes, the CFA showed a better fit: RMSEA of 0.071 (95% CI [0.066, 0.077]), CFI/TLI of 0.955/0.950, SRMR of 0.050, and χ^2^ (108) = 593.861, *p* < 0.001. Overall, this indicates a good model fit, with the RMSEA showing an acceptable fit and the CFI/TLI demonstrating a good model fit. The communalities of the items are acceptable, as shown in Table 4 (ranging from 0.55 to 0.94).

We compared the fit indices between the one-factor model and the four-factor model. As shown in Table 5, the Δχ^2^ between these two models is 5306.55 (6006.50–699.95), which is significantly different from zero (*p* < 0.001). These results indicate that the four-factor model fits the data significantly better than does the one-factor model.

**Table 5:** Results of confirmatory factor analyses: comparison of fit indices between one-factor and four-factor models.

Model	GFI	AGFI	PGFI	TLI	CFI	PNFI	RMSEA	χ^2^	*df*	*p*
One-factor model^a^	0.51	0.38	0.40	0.51	0.54	0.51	0.216	6006.50	120	0.000
Four-factor model^b^	0.93	0.89	0.66	0.95	0.96	0.84	0.071	699.95	108	0.000

GFI, Goodness of Fit Index; AGFI, Adjusted Goodness of Fit Index; PGFI, Parsimony Goodness of Fit Index; TLI, Tucker-Lewis Index; CFI, Comparative Fit Index; PNFI, Parsimony Normed Fit Index; RMSEA, Root Mean Square Error of Approximation.

a All items measuring the four concepts load onto a general factor of recovery experiences.

b Each item loads onto one of four hypothesized factors.

The correlations between the latent factors were all significant at *p* < 0.001 and ranged from 0.30 to 0.65, specifically between psychological detachment and relaxation (0.65), mastery (0.30), and control (0.46); between relaxation and mastery (0.52) and control (0.65); and between mastery and control (0.55). The factor loadings were all significant at *p* < 0.001 and high, ranging from 0.740 to o 0.968.

### 5.2 Internal consistency

We evaluated the internal consistency in the global sample and found that the reliability of the REQ scale is satisfactory, with high internal consistency: psychological detachment (Cronbach *α* = 0.88 and McDonald’s *ω* = 0.89), relaxation (Cronbach α = 0.91 and McDonald’s ω = 0.91), mastery (Cronbach α = 0.90 and McDonald’s ω = 0.90), and control (Cronbach α = 0.91 and McDonald’s ω = 0.91).

### 5.3 Health indicators linked to recovery experiences

Table 6 shows the correlations between all variables (recovery experience dimensions, health indicators, and control variables). Perceived stress and perceived loneliness were negatively related to all the recovery experience dimensions. Physical health, mental health, and sleep quality were positively related to all the recovery experience dimensions. The results of the present study confirm all our initial assumptions.

**Table 6:** Correlations of recovery experience dimensions with sociodemographic variables and potential indicators (*N* = 1,043).

	Measures	Psychological detachment	Relaxation	Mastery	Control
**Some sociodemographics**					
1	Age (years)	0.09**	0.15***	0.12***	0.11***
2	Gender^a^	−0.00	−0.02	−0.08**	−0.06*
3	Life partner^b^	−0.04	−0.01	−0.03	−0.06*
4	Education level^c^	0.03	0.16***	0.14**	0.06*
5	Sector (manufacturing vs. services)^d^	−0.04	−0.03	−0.01	−0.08**
6	Experience (years)	0.03	0.07*	0.06	0.02
7	Weekly workload^e^	−0.26***	−0.24**	−0.05	−0.18***
8	Business size^f^	0.04	0.07*	0.03	0.00
**Potential indicators**					
9	Perceived stress	−0.42***	−0.43***	−0.23***	−0.44***
10	Perceived loneliness	−0.26***	−0.26***	−0.18***	−0.31***
11	Physical health	0.28***	0.41***	0.26***	0.35***
12	Mental health	0.37***	0.44***	0.33***	0.45***
13	Sleep quality	0.28***	0.32***	0.18***	0.30***

*p < 0.05, **p < 0.01, ***p < 0.001.

a Gender was coded as 0 = male and 1 = female.

b Life partner was coded as 0 = yes and 1 = no.

c Education level was coded as 1 = self-taught; 2 = vocational training certificate; 3 = high school diploma; 4 = associate/bachelor’s degree; 5 = master’s degree and 6 = doctorate or higher.

d Sector was coded as 0 = manufacturing and 1 = services.

e Weekly workload was coded as 1 = 40 h or less; 2 = between 40 and 50 h; 3 = between 50 and 60 h; 4 = between 60 and 70 h and 5 = more than 70 h.

f Business size was coded as 0 = less than 10 employees and 1 = more than 10 employees.

## 6 Discussion

The present analysis explored the psychometric properties of the Recovery Experience Questionnaire (REQ) adapted for a sample of French entrepreneurs, a group for whom postwork recovery is crucial but often neglected. Consistent with our assumption and previous studies conducted in other cultural and professional contexts, our CFA supported a four-factor structure (psychological detachment, relaxation, mastery, and control), confirming the factorial validity of the REQ in our sample.

The distinction between the four recovery factors aligns with findings from diverse populations, highlighting the robustness and cross-context applicability of the recovery model by Sonnentag and Fritz (2007). Compared to a single-factor model tested in this study, the four-factor model showed significantly better fit, reinforcing the argument for its adoption to clearly evaluate different aspects of recovery. Additionally, during the CFA analysis, several error covariances were added based on modification indices to improve model fit. These error covariances capture the shared variance between certain items that was not fully explained by the latent factors. Despite this adjustment, the four-factor model remained theoretically coherent and statistically robust, providing further support for the structural validity of the REQ. The Cronbach’s alpha coefficients for each subscale exceeding α = 0.88 demonstrate the excellent internal reliability of the questionnaire. Additionally, each factor (psychological detachment, relaxation, mastery and control) is well represented by appropriate items from the initial model.

Our results revealed significant links between recovery experiences and health indicators such as perceived stress, loneliness at work, physical health, mental health, and sleep quality. These associations align with existing theories positing that good recovery experiences can buffer the deleterious effects of occupational stress and promote physical and mental health. For example, daily recovery experiences, such as feeling recovered in the morning, have been shown to predict better day-level job performance and overall well-being (Binnewies et al., 2009). Interestingly, stress and loneliness were negatively related to all dimensions of recovery, suggesting that the emotional and social burdens of entrepreneurship may hinder individuals’ recovery capacity.

Our findings are consistent with those of similar studies conducted in other countries, which also validated the REQ with a four-factor structure among different professional groups. Several studies have supported the robustness of the four-factor model of recovery experiences across various contexts. For example, Pérez-Nebra et al. (2023), Shimazu et al. (2012), and Kazlauskas et al. (2023) confirmed the four-factor structure, highlighting its applicability in different cultural and professional settings. Taken together, these studies emphasize the universality of the recovery model, although cultural nuances may affect how these factors manifest. The consistency of these findings across diverse populations underscores the reliability and cross-context applicability of the four-factor model in assessing recovery experiences.

While the correlation analyses were conducted on the combined groups, we also performed psychometric invariance analyses to test the validity of the recovery scale across the four surveys of entrepreneurs. These invariance analyses confirmed that the scale measures the same constructs equivalently across the surveys, which validates the use of the overall results. However, it is important to note that psychometric invariance does not guarantee that the relationships between recovery and health indicators are identical across surveys. To explore this issue in more detail, future analyses per survey could identify any potential differences in how recovery and health indicators are related depending on the demographic and professional characteristics of the entrepreneurs.

Given the high levels of stress and the negative impact of loneliness on recovery among entrepreneurs and other professionals (Fernet et al., 2016; Wright and Silard, 2021), the Recovery Experience Questionnaire (REQ) has significant potential in the field of occupational health. The relevance of validating a recovery experience scale in France in particular is underscored by the fact that the country has one of the highest burnout rates among salaried workers in Europe (Future Forum, 2023). Recovery experiences are known to reduce burnout (Song et al., 2021), emphasizing the importance of such a scale not only for entrepreneurs but also for employees. Despite being validated with entrepreneurs, the REQ is a fair starting point for use with salaried workers, providing valuable insights into their recovery processes and assisting in burnout prevention. To support this argument, we conducted the same validation analysis on a sample of 1,231 French agricultural employees. The results reinforced our assumption that the four-factor model is optimal. The exploratory factor analyses revealed four factors with eigenvalues greater than one, and the confirmatory factor analysis (CFA) validated the structural integrity of the four-factor REQ model. The fit indices were strong, with an RMSEA of 0.070 (95% CI [0.065, 0.075]), CFI/TLI of 0.940/0.937, SRMR of 0.064, and χ^2^ (114) = 676.873, *p* < 0.001.

Importantly, we did not modify the items of the Recovery Experience Questionnaire to specifically adapt them to the entrepreneur population. We retained the original scale, which means that any study in French seeking to use a validated version of the Sonnentag and Fritz Recovery Experience Scale can use this version. This approach ensures the comparability of results across different populations and contexts, even if the scale has not been directly validated for their target population.

This study has several limitations. Our study relies solely on correlational analyses. The sample consisted predominantly of entrepreneurs who may face unique stressors and recovery experiences. Future research should aim to include a more diverse range of professional groups to validate the REQ across different occupational contexts. Further research is needed to explore the test–retest reliability of the REQ and its applicability in clinical settings, such as among individuals with high levels of work-related stress or burnout. Examining the psychometric properties of the REQ in diverse professional groups and different cultural contexts will enhance the robustness and applicability of the scale.

## 7 Conclusion

This is the first study presenting a French version of the Recovery Experience Questionnaire (REQ), which exhibits good psychometric properties. We are inclined to name our French version the REQ-F, which is a reliable instrument that can be used to assess recovery experiences among individuals. It was validated via a sample of entrepreneurs but can also be used for salaried workers. The simplicity of the REQ-F makes it suitable for use in both clinical practice and various subclinical research domains. Further studies are needed to confirm its applicability in clinical and subclinical settings. By discriminating between psychological detachment, relaxation, mastery, and control, this tool can significantly contribute to improving occupational health.

## Data Availability

The raw data supporting the conclusions of this article will be made available by the authors, without undue reservation.

## Footnotes

^1^In the present article, we use the term “entrepreneur” to refer to “small business owner.”
