# Supplementary material for: Validation and psychometric evaluation of the French version of the recovery experience questionnaire: internal consistency and validity assessment
Source: Front Psychol. 2024 Oct 2;15:1466905. doi: 10.3389/fpsyg.2024.1466905 (PMC11480058; doi:10.3389/fpsyg.2024.1466905)
Supplement: Supplementary file 1 [file Table_1.docx]

Supplementary Material 1

Means, Standard Deviations, Cronbach's Alpha, and Correlations of Variables Used in the Study (N = 1,043)

|  | Measures | Mean | SD | 1 | 2 | 3 | 4 | 5 | 6 | 7 | 8 |  |
| --- | --- | --- | --- | --- | --- | --- | --- | --- | --- | --- | --- | --- |
| Demographics | | |  |  |  |  |  |  |  |  |  |  |
| 1 | Age (years) | 50.00 | 9.65 | (n.a.) |  |  |  |  |  |  |  |  |
| 2 | Gender^a)^ | 0.49 | 0.50 | –0.14*** | (n.a.) |  |  |  |  |  |  |  |
| 3 | Life partner^b)^ | 3.00 | 1.49 | 0.05 | 0.14*** | (n.a.) |  |  |  |  |  |  |
| 4 | Education level^c)^ | 3.70 | 1.36 | 0.04 | 0.12*** | –0.06 | (n.a.) |  |  |  |  |  |
| 5 | Sector (manufacturing vs services)^d)^ | 0.83 | 0.38 | –0.09** | 0.22*** | 0.18*** | 0.00 | (n.a.) |  |  |  |  |
| 6 | Experience (years) | 13.69 | 9.20 | 0.49*** | –0.08* | 0.09** | 0.03 | –0.08* | (n.a.) |  |  |  |
| 7 | Weekly workload^e)^ | 2.79 | 1.20 | –0.05 | –0.21*** | 0.05 | 0.04 | 0.01 | –0.06* | (n.a.) |  |  |
| 8 | Business size | 0.18 | 0.39 | 0.08* | –0.10** | –0.01 | 0.19*** | –0.06 | 0.13*** | 0.17*** | (n.a.) |  |
| Recovery experiences | | |  |  |  |  |  |  |  |  |  |  |
| 9 | Psychological detachment | 2.28 | 1.01 | 0.09** | 0.00 | –0.05 | 0.03 | –0.04 | 0.03 | –0.26*** | 0.04 |  |
| 10 | Relaxation | 2.80 | 1.10 | 0.15*** | –0.02 | –0.01 | 0.16*** | –0.03 | 0.07* | –0.24*** | 0.07* |  |
| 11 | Mastery | 2.93 | 1.10 | 0.12*** | –0.08** | –0.03 | 0.14*** | –0.01 | 0.06 | –0.05 | 0.03 |  |
| 12 | Control | 3.26 | 1.12 | 0.11*** | –0.06* | –0.06* | 0.06* | –0.08** | 0.02 | –0.18*** | 0.00 |  |
| Potential indicators | | |  |  |  |  |  |  |  |  |  |  |
| 13 | Perceived stress | 3.47 | 0.94 | –0.16*** | 0.78* | 0.05 | 0.05 | 0.04 | –0.01 | 0.32*** | 0.09* |  |
| 14 | Perceived loneliness | 3.24 | 1.19 | –0.04 | 0.04 | 0.05 | 0.01 | –0.03 | –0.02 | 0.08* | –0.07* |  |
| 15 | Physical health | 2.92 | 1.04 | 0.09** | –0.08* | –0.11*** | 0.07* | –0.06* | 0.04 | –0.15*** | –0.01 |  |
| 16 | Mental health | 2.86 | 1.09 | 0.17*** | –0.12*** | –0.06 | 0.07* | –0.08* | 0.06 | –0.07* | 0.02 |  |
| 17 | Sleep quality | 2.57 | 1.16 | 0.08** | –0.10*** | 0.03 | 0.10*** | –0.04 | 0.04 | –0.08* | 0.00 |  |

*Note.* * p < .05, ** p < .01, *** p < .001

|  | Measures | 9 | 10 | 11 | 12 | 13 | 14 | 15 | 16 | 17 |
| --- | --- | --- | --- | --- | --- | --- | --- | --- | --- | --- |
| Demographics | |  |  |  |  |  |  |  |  |  |
| 1 | Age (years) |  |  |  |  |  |  |  |  |  |
| 2 | Gender^a)^ |  |  |  |  |  |  |  |  |  |
| 3 | Life partner^b)^ |  |  |  |  |  |  |  |  |  |
| 4 | Education level^c)^ |  |  |  |  |  |  |  |  |  |
| 5 | Sector (manufacturing vs services)^d)^ |  |  |  |  |  |  |  |  |  |
| 6 | Experience (years) |  |  |  |  |  |  |  |  |  |
| 7 | Weekly workload^e)^ |  |  |  |  |  |  |  |  |  |
| 8 | Business size |  |  |  |  |  |  |  |  |  |
| Recovery experiences | |  |  |  |  |  |  |  |  |  |
| 9 | Psychological detachment | (0.88) |  |  |  |  |  |  |  |  |
| 10 | Relaxation | 0.60*** | (0.91) |  |  |  |  |  |  |  |
| 11 | Mastery | 0.27*** | 0.43*** | (0.90) |  |  |  |  |  |  |
| 12 | Control | 0.43*** | 0.56*** | 0.48*** | (0.91) |  |  |  |  |  |
| Potential indicators | |  |  |  |  |  |  |  |  |  |
| 13 | Perceived stress | –0.42*** | –0.43*** | –0.23*** | –0.44*** | (n.a.) |  |  |  |  |
| 14 | Perceived loneliness | –0.26*** | –0.26*** | –0.18*** | –0.31*** | 0.34*** | (n.a.) |  |  |  |
| 15 | Physical health | 0.28*** | 0.41*** | 0.26*** | 0.35*** | –0.39*** | –0.31*** | (n.a.) |  |  |
| 16 | Mental health | 0.37*** | 0.44*** | 0.33*** | 0.45*** | –0.50*** | –0.38*** | 0.57*** | (n.a.) |  |
| 17 | Sleep quality | 0.28*** | 0.32*** | 0.18*** | 0.30*** | –0.41*** | –0.27*** | 0.47*** | 0.50*** |  |

*Note.* * p < .05, ** p < .01, *** p < .001. ^a)^ Gender was coded as 0 = male and 1 = female. ^b)^ Life partner was coded as 0 = yes and 1 = no. ^c)^ Education level was coded as 1 = self-taught; 2 = vocational training certificate; 3 = high school diploma; 4 = associate/bachelor’s degree; 5 = master’s degree and 6 = doctorate or higher. ^d)^ Sector was coded as 0 = manufacturing and 1 = services. ^e)^ Weekly workload was coded as 1 = 40 hours or less; 2 = between 40 and 50 hours; 3 = between 50 and 60 hours; 4 = between 60 and 70 hours and 5 = more than 70 hours.
