# Supplementary material for: Validation and psychometric evaluation of the French version of the recovery experience questionnaire: internal consistency and validity assessment
Source: Front Psychol. 2024 Oct 2;15:1466905. doi: 10.3389/fpsyg.2024.1466905 (PMC11480058; doi:10.3389/fpsyg.2024.1466905)
Supplement: Supplementary file 2 [file Table_2.DOCX]

Supplementary Material 2

Residual Covariances and Their Significance

| Covariance | Estimate | Std.Error | Z-value | P-value |
| --- | --- | --- | --- | --- |
| Item 1 ~~ Item 2 | 0.228 | 0.029 | 7.751 | < 0.001 |
| Item 3 ~~ Item 4 | –0.132 | 0.034 | –3.828 | < 0.001 |
| Item 1 ~~ Item 6 | 0.014 | 0.010 | 1.378 | 0.168 |
| Item 6 ~~ Item 7 | 0.149 | 0.023 | 6.472 | < 0.001 |
| Item 10 ~~ Item 11 | 0.145 | 0.024 | 5.922 | < 0.001 |
| Item 13 ~~ Item 14 | 0.109 | 0.022 | 5.006 | < 0.001 |
